# Cholesterol-Enhanced Polylactide-Based Stereocomplex Micelle for Effective Delivery of Doxorubicin

**DOI:** 10.3390/ma8010216

**Published:** 2015-01-12

**Authors:** Jixue Wang, Weiguo Xu, Jianxun Ding, Shengfan Lu, Xiaoqing Wang, Chunxi Wang, Xuesi Chen

**Affiliations:** 1 Department of Urology, the First Hospital of Jilin University, Changchun 130021, China; E-Mails: wangjx@ciac.ac.cn (J.W.); chunxi_wang@126.com (C.W.); 2 Key Laboratory of Polymer Ecomaterials, Changchun Institute of Applied Chemistry, Chinese Academy of Sciences, Changchun 130022, China; E-Mails: wgxu@ciac.ac.cn (W.X.); sflu@ciac.ac.cn (S.L.); xschen@ciac.ac.cn (X.C.)

**Keywords:** cholesterol, controlled delivery, doxorubicin, malignancy therapeutics, polylactide, stereocomplex micelle

## Abstract

Nanoscale micelles as an effective drug delivery system have attracted increasing interest in malignancy therapy. The present study reported the construction of the cholesterol-enhanced doxorubicin (DOX)-loaded poly(D-lactide)-based micelle (CDM/DOX), poly(L-lactide)-based micelle (CLM/DOX), and stereocomplex micelle (CSCM/DOX) from the equimolar enantiomeric 4-armed poly(ethylene glycol)–polylactide copolymers in aqueous condition. Compared with CDM/DOX and CLM/DOX, CSCM/DOX showed the smallest hydrodynamic size of 96 ± 4.8 nm and the slowest DOX release. The DOX-loaded micelles exhibited a weaker DOX fluorescence inside mouse renal carcinoma cells (*i.e.*, RenCa cells) compared to free DOX·HCl, probably because of a slower DOX release. More importantly, all the DOX-loaded micelles, especially CSCM/DOX, exhibited the excellent antiproliferative efficacy that was equal to or even better than free DOX·HCl toward RenCa cells attributed to their successful internalization. Furthermore, all of the DOX-loaded micelles exhibited the satisfactory hemocompatibility compared to free DOX·HCl, indicating the great potential for systemic chemotherapy through intravenous injection.

## 1. Introduction

Despite the considerable advances in the treatment of malignancy, the mortality rate of malignancy remains relatively high all over the world [1]. Chemotherapy plays a key role in all the approaches for malignancy therapy [2]. However, the traditional chemotherapy drugs always cause various side effects, which limit the clinical use of chemotherapy [3]. With the rapid development of nanotechnology, the nanomedicines based on polymeric nanoparticles probably hold the key to address the drawbacks of traditional chemotherapy drugs and achieve a better antitumor efficacy. Recently, there are numerous nanoscale drug delivery systems (DDSs), such as liposomes [4,5], micelles [6,7], nanogels [8,9], and so on. Among of them, micelles have got more and more attention due to their excellent conjugation or entrapment of drugs and effective accumulation around the tumor cells through the enhanced permeability and retention (EPR) effect [10].

Doxorubicin (DOX) as an antitumor drug is widely used in clinical chemotherapy. However, DOX exists serious dose-dependent toxicity, because it cannot show a therapeutic action unless it enters into cell nucleus, which will induce serious side effects on the body [11]. In order to solve these problems, the advanced DDSs are in urgent need of development. An ideal DDS should be able to transport the drug (e.g., DOX) into cell effectively and maintain an effective drug concentration in a long time. Furthermore, an excellent DDS should not exhibit obvious toxic effects on normal organs or tissues [12].

Polylactide (PLA) has several stereoisomeric structures, including poly(D-lactide) (PDLA), poly(L-lactide) (PLLA), and poly(D,L-lactide) (PDLLA) [13]. PLA is a biodegradable and biocompatible polymer, which is widely used in the realms of drug delivery, bioengineering, and so on [14,15,16,17]. However, the high hydrophobicity, low drug loading efficiency, and long degradation time have limited the biomedical application of PLA [18]. Poly(ethylene glycol) (PEG), a polyether, has lots of advantages, such as good hydrophilicity, efficient resistance to immunological recognition, favorable biocompatibility, and so on [19,20,21], which makes it be widely applied in medicine. Since PEG was used to prolong the circulation time of liposome in 1990 [22], the nanoparticles modified with PEG have been widely investigated [23,24,25,26]. Among them, the biodegradable block copolymers, which are constructed by PEG and PDLA, PLLA, or PDLLA, exhibit fascinating potential for formulating DDSs [27,28,29]. Ouahab* et al.* constructed the pH-sensitive charge-reversal and cell penetrating peptides-conjugated PEG–PLA micelles for docetaxel delivery, which showed satisfactory antitumor efficiency* in vitro* [30]. Chen and coworkers prepared the stereocomplex micelles (SCMs) of enantiomeric PEG–PLA block copolymers to delivery rifampin, which showed enhanced stability in water and higher encapsulation efficiencies compared to single PEG–PDLA and PEG–PLLA micelles [31]. Liu and colleagues fabricated a DOX-loaded 4-armed SCM that exhibited better antitumor effect than the micelles with single component [32]. Moreover, Genexol^®^-PM composed of PEG and PDLLA is the only clinically approved nanoscale polymeric chemotherapeutic, which was developed by Samyang Genex Co. (Seoul, Korea) [33,34]. Cholesterol is an essential component of cell membrane and has an important influence on its function [35]. Cholesterol can be incorporated into the DDSs to facilitate the cellular uptake, which should be attributed to its excellent structural compatibility with cell membrane [36,37]. There are lots of researches about the influence of cholesterol on DDSs, which show some positive effects on the nanosized drug carriers [38,39]. 

**Scheme 1:**
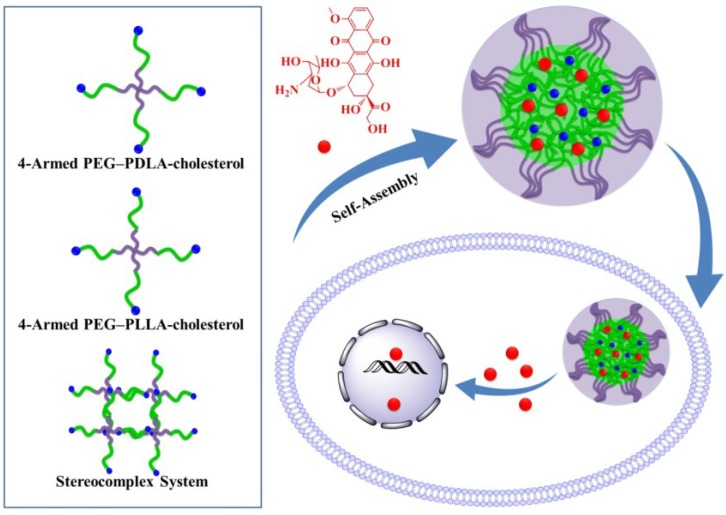
Schematic illustration for fabrication of CDM/DOX, CLM/DOX, and CSCM/DOX, and their cellular uptakes by RenCa cells* in vitro*.

In this work, DOX was loaded into the cholesterol-mediated 4-armed PEG–PDLA-, PEG–PLLA-, and equimolar PEG–PDLA/PEG–PLLA-based micelles, noted as CDM/DOX, CLM/DOX, and CSCM/DOX, respectively, by nanoprecipitation (Scheme 1). The formulations, especially CSCM/DOX, exhibited excellent antiproliferative activities toward RenCa cells (a mouse renal carcinoma cell line), which were even better than free DOX·HCl. In addition, the DOX-loaded micelles showed decreased hemolysis rates compared with free DOX·HCl, which endowed them with great potential for* in vivo* applications.

## 2. Results and Discussion

### 2.1. Fabrication and Characterization of DOX-Loaded Micelles

**Figure 1:**
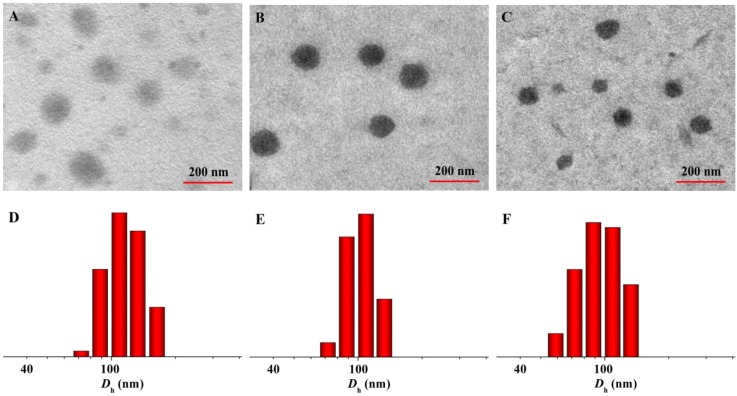
Typical TEM micrographs (**A**–**C**) and *D*_h_s (**D**–**F**) of CDM/DOX (**A** and **D**), CLM/DOX (**B** and **E**), and CSCM/DOX (**C** and **F**).

**Figure 2:**
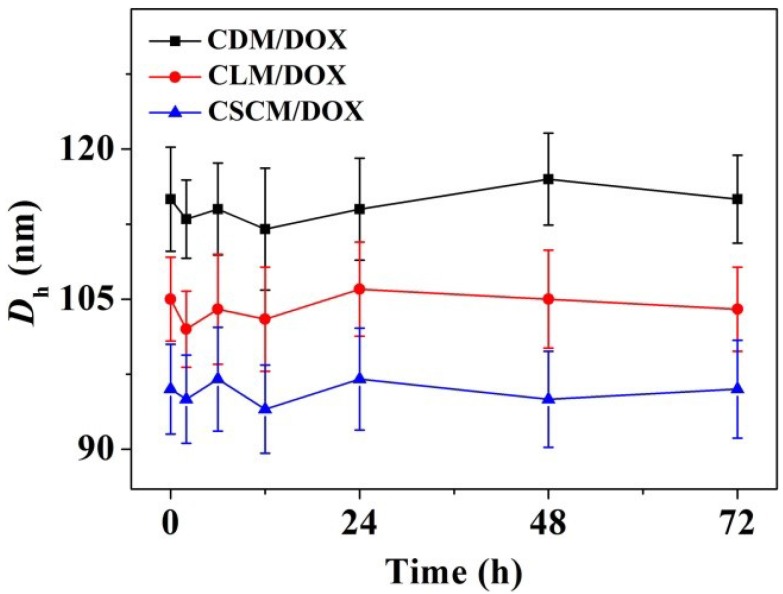
*D*_h_ changes of CDM/DOX, CLM/DOX, and CSCM/DOX* versus* time in PBS at pH 7.4, 25 °C. Each set of data was presented as mean ± standard deviation (SD) (*n* = 3).

The branched and multi-armed copolymers have attracted great attention for their more excellent rheological, mechanical, and biomedical properties compared to the linear one and have been widely explored [40]. In this work, pairs of enantiomeric cholesterol-modified 4-armed copolymers of PEG–PDLA and PEG–PLLA were employed as matrices for controlled antitumor drug delivery. The amphiphilic nature of 4-armed PEG–PLA-cholesterol copolymers made them easy to self-organize into micelles in aqueous solution. As shown in Scheme 1, DOX was physically encapsulated by CDM, CLM, or CSCM by nanoprecipitation, yielding CDM/DOX, CLM/DOX, and CSCM/DOX, respectively. The drug-loading contents (DLCs) of CDM/DOX, CLM/DOX, and CSCM/DOX were calculated to be 8.3, 8.8, and 9.5 wt.% and the drug-loading efficiencies (DLEs) were 45.1, 48.2, and 52.2 wt.%, respectively. Compared to CDM/DOX and CLM/DOX, CSCM/DOX exhibited the highest DLC and DLE because of the stable stereocomplex crystallization of enantiomeric PLA in micellar core [31]. The successful preparation of various DOX-loaded micelles was confirmed by transmission electron microscopy (TEM) and dynamic laser scattering (DLS). As shown in Figure 1A–C, all the laden micelles were well dispersed with clear spherical morphologies. The apparent mean diameters of CDM/DOX, CLM/DOX, and CSCM/DOX were around 100, 90, and 80 nm, respectively. In comparison, the hydrodynamic diameters (*D*_h_s) of these micelles tested by DLS were 118 ± 5.2, 104 ± 4.3, and 96 ± 4.8 nm, respectively (Figure 1D–F). Furthermore, the polydispersity indices (PDIs) of *D*_h_s were calculated to be 0.14, 0.14, and 0.13, respectively, which indicated the narrow size distribution of loading micelles. The sizes of DOX-loaded micelles measured by DLS were larger than those detected by TEM mainly because of the hydration state of micelles in DLS tests [41]. The particle size is an important factor for DDSs to deliver drug into tumor effectively. The appropriate sizes of these micelles might give them with excellent capability for selective accumulation in tumor through the EPR effect [10]. Moreover, as shown in Figure 2, the laden micelles showed excellent stability for at least 72 h incubated in phosphate-buffered saline (PBS) at pH 7.4.

### 2.2. DOX Release from Various Formulations

**Figure 3:**
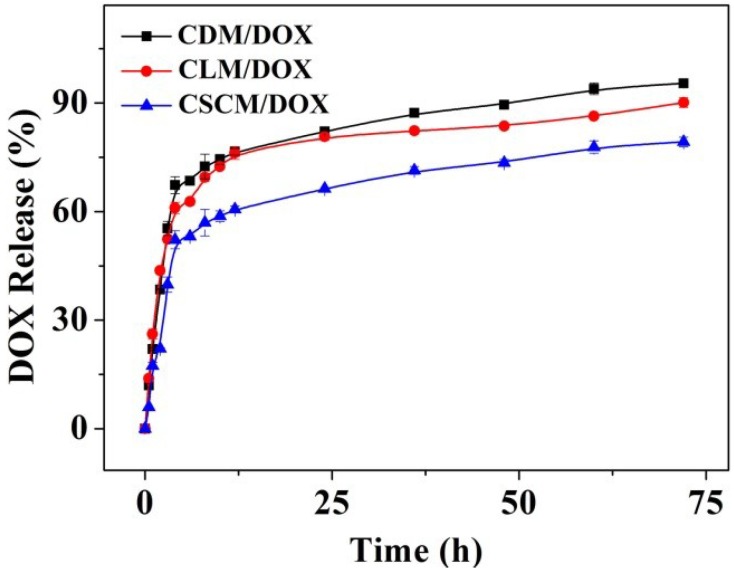
Release behaviors of CDM/DOX, CLM/DOX, and CSCM/DOX in PBS at pH 7.4, 37 °C. Each set of data was presented as mean ± SD (*n* = 3).

The* in vitro* release behaviors of these micelles were examined in PBS at pH 7.4, imitating the conditions in normal physiological tissues. As shown in Figure 3, CDM/DOX and CLM/DOX showed a similar release behavior, which presented three phases: an initial burst release stage, in which 65% of the loaded DOX was released during 6 h; a continuous slow release phase, in which 85% of the loaded DOX was released in a continuous way during 36 h; a platform period, in which only a little loaded DOX was released until 72 h. The initial burst release might be attributed to the absorption of DOX by the shallow parts of the micelles. The mechanism of slow release might be related to the diffusion of DOX through the micelles and the degradation of PLA block [42]. Interestingly, CSCM/DOX exhibited slower drug release than those with single component because of the enhanced stability of CSCM [43]. As time was extended to 36 h, the DOX release of all these laden micelles was tended to be stable. In addition, the cumulative DOX release of these two micelles with single component was about 90 wt.% in 72 h, while that of CSCM/DOX was about 75 wt.%. The results showed that these micelles, especially CSCM, could load and controllably release DOX effectively.

**Figure 4:**
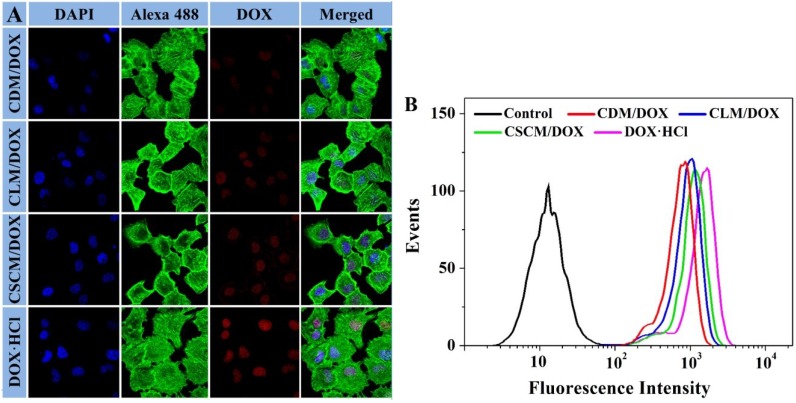
Typical CLSM microimages (**A**) and FCM determinations (**B**) of RenCa cells incubated with CDM/DOX, CLM/DOX, CSCM/DOX, or free DOX·HCl for 2 h.

Furthermore, the cellular uptakes and intracellular release behaviors of these DOX-loaded micelles were explored on RenCa cells through confocal laser scanning microscopy (CLSM) and flow cytometry (FCM). As shown in CLSM microimages (Figure 4A), the fluorescence intensity of cells co-cultured with free DOX·HCl was higher than those of DOX-loaded micelles for 2 h. It might because that the cellular uptake of free DOX·HCl by diffusion was quicker than those of DOX-incorporated micelles by endocytosis [44]. Moreover, the fluorescence intensity of DOX in CSCM/DOX group was higher than those of CDM/DOX and CLM/DOX groups, which was possibly attributed to the slower extracellular DOX release and more efficient DOX release in intracellular condition [32]. For further confirmation, the FCM histograms of DOX-loaded micelles and free DOX·HCl were performed and shown in Figure 4B. RenCa cells without any treatments served as blank control, which showed only the autofluorescence of cells. The signal intensity of CDM/DOX and CLM/DOX made little difference. The fluorescence intensity of CSCM/DOX in the nuclei was higher than those of CDM/DOX and CLM/DOX, and free DOX·HCl exhibited the highest fluorescence intensity. The results of FCM were agreed very well with that of CLSM, which showed the effective internalization of DOX-loaded micelles by RenCa cells.

### 2.3. In Vitro Assessment of Cell Viability

**Figure 5:**
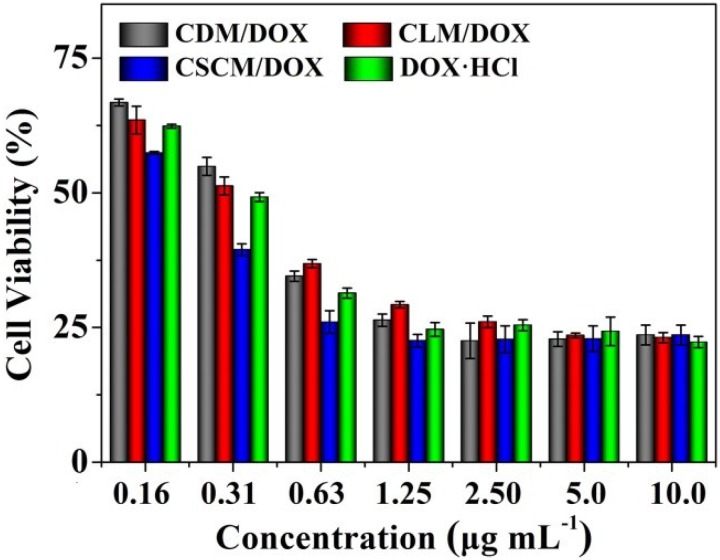
Relative cell viabilities of DOX-loaded micelles toward RenCa cells with free DOX·HCl as control. Each set of data was presented as mean ± SD (*n* = 4).

In order to evaluate the toxicity profiles of DOX-loaded micelles and free DOX·HCl, the cell viability of RenCa cells was evaluated by a 3-(4.5-dimethyl-thiazol-2-yl)-2.5-diphenyl tetrazolium bromide (MTT) assay. The cellular proliferation inhibition capabilities of DOX-loaded micelles and free DOX·HCl were compared. As shown in Figure 5, CDM/DOX and CLM/DOX appeared lower proliferation inhibitory efficacy than free DOX·HCl at equivalent DOX concentration after incubation for 48 h. The phenomenon might be due to the exist of cholesterol, which enter the cells possibly through a low-density lipoprotein receptor-mediated endocytosis pathway [45,46] and the high-energy dependency of the endocytosis process [47]. However, CSCM/DOX exhibited more effective proliferation inhibition effects on RenCa cells than those of CDM/DOX, CLM/DOX, and free DOX·HCl. It might be relevant to the greater stability of SCM, so less cholesterol appeared on the surface of CSCM/DOX, which less influenced endocytosis. What's more, the improved stability of the micelle meant the less extracellular drug release. Therefore, a greater amount of drug was internalized into the cells by endocytosis and sustained drug release in tumor cells effectively. The half maximal inhibitory concentrations (IC_50_) of CDM/DOX, CLM/DOX, CSCM/DOX, and free DOX·HCl were calculated to be 0.44, 0.37, 0.22, and 0.33 μg mL^−1^, respectively. The lowest IC_50_ of CSCM/DOX quantitatively showed its enhanced antiproliferative capability against the tumor cells and the potential advantage to be used as a potential antitumor drug formulation.

### 2.4. Evaluations of Serum Albumin-Tolerance Stability and Hemocompatibility

**Figure 6:**
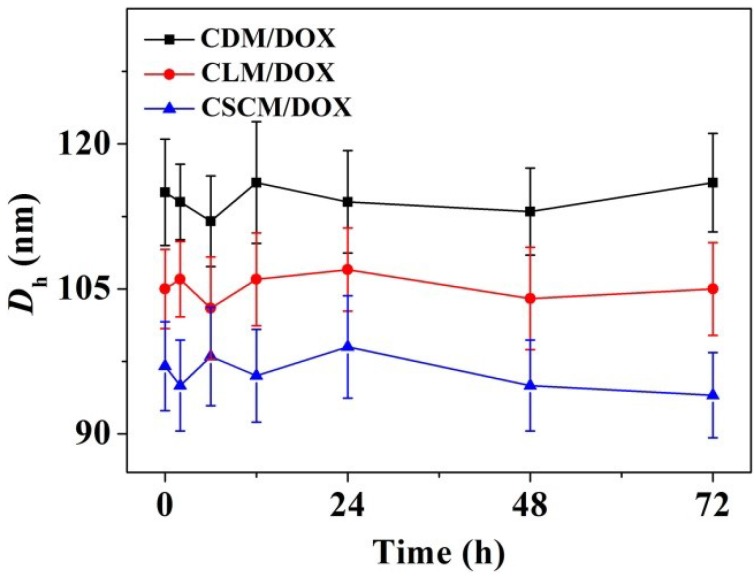
*D*_h_ changes of CDM/DOX, CLM/DOX, and CSCM/DOX* versus* time in PBS-buffered BSA solution (30.0 mg mL^−1^) at pH 7.4, 25 °C. Each set of data was presented as mean ± SD (*n* = 3).

The evaluations of serum albumin-tolerance stability and hemocompatibility of laden micelles are necessary because the corresponding formulations are designed to be finally administrated via intravenous injection [48]. In this study, the stability of these DOX-loaded micelles incubated in PBS-buffered bovine serum albumin (BSA) solution (30.0 mg mL^−1^) was tested by DLS at 25 °C. As shown in Figure 6, all the laden micelles exhibited excellent stability during the measurement of 72 h. Moreover, the *D*_h_s of these laden micelles in PBS with BSA were similar to the above results in PBS without BSA (Figure 2). It indicated that the DOX-loaded micelles kept excellent stability in BSA solution.

**Figure 7:**
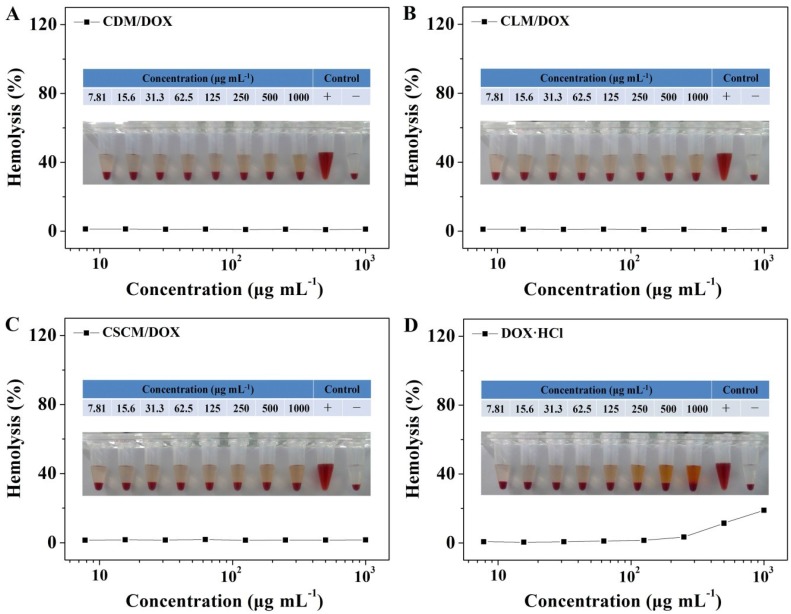
Hemolysis behaviors of CDM/DOX (**A**); CLM/DOX (**B**); CSCM/DOX (**C**); and free DOX·HCl (**D**).

As mentioned above, the excellent hemocompatibility is an important precondition of DOX-loaded micelles for the final application in clinic through the intravenous injection, which is the main administration approach for most drug delivery systems [49]. As shown in Figure 7, the hemolytic activities of these micelles and free DOX·HCl were tested by a spectrophotometry approach. The profiles revealed that these micelles were almost no obvious hemolysis activities with DOX·HCl concentrations up to 1.0 mg mL^−1^, while free DOX·HCl showed more serious hemolysis of red blood cells (RBCs). The results demonstrated that the DOX-loaded micelles had satisfactory blood compatibility.

## 3. Experimental Section

### 3.1. Materials

4-Armed PEG with a number-average molecular weight of 10,000 Da was purchased from Shanghai Seebio Biotech, Inc. (Shanghai, China) and used as received. DLA and LLA were provided by Changchun SinoBiomaterials Co., Ltd. (Changchun, China) and recrystallized from ethyl acetate under argon atmosphere before use. 4-Armed PEG–PDLA and PEG–PLLA were synthesized as our previously reported proposal [32]. In briefly, 10.0 g (1.0 mmol) of 4-armed PEG were azeotropically distilled with 200.0 mL of toluene at 120 °C to remove traces of water. And then, 5.8 g (40 mmol) of DLA or LLA and 100.0 mL of dried toluene were added into the PEG solution. The reaction was performed at 110 °C for 24 h. After the reaction, the copolymer was precipitated in 500.0 mL of diethyl ether. Then the product was dissolved in dichloromethane and precipitated in diethyl ether again. This operation was repeated three times. The obtained product was filtered and dried *in vacuum* overnight. The degree of polymerization (DP) of PLA in each arm was calculated to be 16 based on the data of proton nuclear magnetic resonance. The number-average molecular weight (*M*_n_) of copolymer was estimated to be 14,600 g mol^−1^. The cholesterol-modified copolymers were prepared through the condensation reaction between 4-armed PEG–PLA and cholesteryl chloroformate [50]. Cholesteryl chloroformate, MTT, 4′,6-diamidino-2-phenylindole (DAPI), Alexa Fluor 488 phalloidin (Alexa 488), and BSA were purchased from Sigma-Aldrich (Shanghai, China). Doxorubicin hydrochloride (DOX·HCl) was obtained from Beijing HuaFeng United Technology Co., Ltd. (Beijing, China). Clear 6-well and 96-well tissue culture polystyrene (TCP) plates were obtained from Corning Costar Co. (Cambridge, MA, USA). The deionized water was prepared through Milli-Q water purification equipment (Millipore Co., Milford, MA, USA).

### 3.2. DOX Encapsulation

DOX was loaded into micelles through a nanoprecipitation method [51]. In briefly, DOX·HCl (21.3 mg) were dissolved in 6.0 mL of Milli-Q water, and then were slowly added into 10.0 mL of 4-armed PLLA–PEG-cholesterol copolymer solution in *N*,*N*-dimethylformamide (DMF) (10.0 mg mL^−1^). After that, 2.0 mL of PBS was added into the mixed solution. It was continuous stirring at room temperature for 12 h and subsequently dialyzed against deionized water for 12 h (molecular weight cut-off (MWCO) = 3500 Da). At last, the CLM/DOX was obtained by lyophilisation. Both CDM/DOX and CSCM/DOX were fabricated by the same protocol.

In order to detect the DLC and DLE, the DOX-loaded micelles were dissolved in DMF and stirred for 12 h at room temperature. After that, the amount of DOX in micelles were detected by the fluorescence spectroscopy with a standard curve method on a Fluorescence Master System with software Felix 4.1.0 (λ_ex_ = 480 nm; Photon Technology International, Inc., Lawrenceville, NJ, USA). The DLC and DLE of DOX-loaded micelles were calculated by Equations (1) and (2), respectively.

(1) $$\text{DLC(wt}.\%)=\frac{\text{weight of drug in micelle}}{\text{weight of drug-loaded micelle}}\times 100$$

(2) $$\text{DLE(wt}.\%)=\frac{\text{weight of drug in micelle}}{\text{total weight of feeding drug}}\times 100$$

### 3.3. Measurements

At the predetermined times after the dissolution of DOX-loaded micelles in PBS at pH 7.4, the *D*_h_s of laden micelles were determined using DLS at 25 °C on a WyattQELS apparatus. The intensity results were obtained, and the average histograms were given. The PDI of *D*_h_ was defined as the ratio of standard deviation and mean of *D*_h_. TEM experiments were carried out on a JEOL JEW-1011 instrument operating at an accelerating voltage of 100 kV. 10.0 μL of loading micelle solution (0.1 mg mL^−1^) was dipped on a copper grid and then dried at room temperature in the air.

### 3.4. In Vitro DOX Release

The release profiles of DOX-loaded micelles were assessed in PBS at pH 7.4, a mimicking normal physiological condition. In brief, 1.0 mg of CDM/DOX, CLM/DOX, or CSCM/DOX was dissolved in 10.0 mL PBS and then transferred into a dialysis bag (MWCO = 3500 Da). After that, the dialysis bag was put into a beaker and subsequently 100.0 mL of PBS was added at 37 °C with continuous vibrations of 70 rpm. At the predetermined time points, 2.0 mL of release medium was taken out for test, and an equal volume of fresh PBS was added into the beaker. The amount of released DOX was determined using fluorescence spectroscopy.

### 3.5. Intracellular DOX Release Analyses

The abilities of micelles to transport DOX into RenCa cells were qualitatively detected by CLSM and quantitatively estimated by FCM.

#### 3.5.1. CLSM

The cells were seeded on glass coverslips in 6-well plates at a density of 2.0 × 10^5^ cells per well in 2.0 mL of complete high glucose Dulbecco’s modified Eagle’s medium (HG-DMEM), and cultured at 37 °C for 24 h. CDM/DOX, CLM/DOX, CSCM/DOX, or free DOX·HCl was added to each well with a final DOX·HCl concentration of 10.0 μg mL**^−^**^1^. After co-incubation for 2 h, the medium was removed and the cells on glass coverslips were washed with PBS five times. After that, the immobilization was executed with 4% (w/v) PBS-buffered paraformaldehyde for 20 min at room temperature. And then, the cells were washed with PBS five times, and added in 0.1% (v/v) Triton X-100 in PBS for 12 min at room temperature. After being washed with PBS five times, the cells were subsequently stained with DAPI for 3 min. Afterwards, the cells were washed with PBS five times. At last, the filamentous actin was dyed with Alexa 488 for 30 min at 37 °C, and washed with PBS five times. The CLSM microimages of cells were photographed by a LSM 780 CLSM (λ_ex_ = 488 nm; Carl Zeiss, Jena, Germany).

#### 3.5.2. FCM

RenCa cells were seeded in 6-well plates at a density of 2.0 × 10^5^ cells per well and cultured with 2.0 mL of complete HG-DMEM for 24 h. And then, CDM/DOX, CLM/DOX, CSCM/DOX, or free DOX·HCl was added to each well with a final DOX·HCl concentration of 10.0 μg mL^−1^. Cells without treatment were used as control. After co-culture for 2 h, the medium was removed and the cells were washed with PBS five times. After that, all the cells were digested by trypsin, suspended in PBS, and centrifuged at 3500 rpm for 5 min. The supernatant was discarded and the bottom cells were resuspended in 0.3 mL of PBS. Data was analyzed by a flow cytometer (λ_ex_ = 488 nm; Beckman, CA, USA).

### 3.6. Cytotoxicity Assays

The cytotoxicities of DOX-loaded micelles and free DOX·HCl with a DOX·HCl concentration from 0.16 to 10.0 μg mL^−1^ were conducted toward RenCa cells by a MTT assay. In brief, 180.0 μL of cell suspension containing 8.0 × 10^3^ cells in complete HG-DMEM was planted into 96-well plates and incubated at 37 °C for 24 h. And then, various DOX formulations in 20.0 μL of PBS were added to each well and cultured for another 48 h. Subsequently, 20.0 μL of MTT at a concentration of 5.0 mg mL^−1^ was added and incubated for further 4 h. After that, the medium was carefully removed, and 150.0 μL of dimethyl sulfoxide (DMSO) was added to each well to dissolve the MTT formazan generated by the live cells. The plates were vibrated for 5 min before detection. The absorbance of medium was measured at 490 nm using a Bio-Rad 680 microplate reader. The cell viability was calculated as Equation (3).

(3) $$\text{Cell Viability (\%)}=\frac{A\text{sample}}{A\text{control}}\times 100$$

In Equation (3), the *A*_sample_ and *A*_control_ represented the absorbances of sample and control wells, respectively.

### 3.7. Serum Albumin-Tolerance Stability Assays

The stability of DOX-loaded micelles in PBS-buffered BSA solution (30 mg mL^−1^) at pH 7.4, 25 °C, was tested by DLS at different time points.

### 3.8. Hemolysis Activity Tests

The hemolytic activity properties of CDM/DOX, CLM/DOX, CSCM/DOX, and free DOX·HCl were evaluated by a spectrophotometry technique. Typically, the fresh rabbit blood was obtained from the Experimental Animal Center of Jilin University, and then the blood was stabilized with dipotassium ethylene diamine tetraacetate in normal saline (NS). The blood was centrifuged at 1500 rpm for 10 min in order to separate the RBCs. Then the obtained RBCs were carefully washed and diluted. Next, CDM/DOX, CLM/DOX, CSCM/DOX, and free DOX·HCl at different concentrations were added in the suspended RBCs at 37 °C for 2 h. NS was used as negative control and Triton X-100 (*i.e.*, a lysing agent of RBCs) was used as positive control. After that, the RBCs were separated at 3000 rpm for 10 min, and 180.0 μL of supernatant of each sample was collected and added into a 96-well plate. Then the free hemoglobin in the supernatant was tested using a Bio-Rad 680 microplate reader at 570 nm. The hemolytic ratio of RBCs was calculated as Equation (4).

(4) $$\text{Hemolytic Ratio(\%)}=\frac{A_{\text{sample}}-A_{\text{negative control}}}{A_{\text{positive control}}-A_{\text{negatice control}}}\times 100$$

In Equation (4), the *A*_sample_, *A*_negative control_, and *A*_positive control_ represented the absorbances of sample, and negative and positive controls, respectively.

## 4. Conclusions

In summary, CDM/DOX, CLM/DOX, and CSCM/DOX were constructed with diameters at ~100 nm, which exhibited a proper size for the selective accumulation in tumor tissue through the EPR effect. Compared to CDM/DOX and CLM/DOX composed with a single polymeric component, CSCM/DOX showed smaller particle size and slower DOX release. What's more, all these DOX-loaded micelles, especially CSCM/DOX, could be effectively internalized by RenCa cells. More importantly, CSCM/DOX showed a higher antiproliferative activity on RenCa cells than both CDM/DOX and CLM/DOX, and even free DOX·HCl after incubation for 48 h. In addition, all the DOX-loaded micelles showed satisfactory biocompatibility compared with free DOX·HCl. All in all, the CSCM is probably used as an effective drug delivery system in the clinical chemotherapy of malignancy.

## References

[1] Shi F.H., Ding J.X., Xiao C.S., Zhuang X.L., He C.L., Chen L., Chen X.S. (2012). Intracellular microenvironment responsive PEGylated polypeptide nanogels with ionizable cores for efficient doxorubicin loading and triggered release. J. Mater. Chem..

[2] Hao Y.B., Yi S.Y., Ruan J., Zhao L., Nan K.J. (2014). New insights into metronomic chemotherapy-induced immunoregulation. Cancer Lett..

[3] Malam Y., Loizidou M., Seifalian A.M. (2009). Liposomes and nanoparticles: Nanosized vehicles for drug delivery in cancer. Trends Pharmacol. Sci..

[4] Koren E., Apte A., Jani A., Torchilin V.P. (2012). Multifunctional PEGylated 2C5-immunoliposomes containing pH-sensitive bonds and TAT peptide for enhanced tumor cell internalization and cytotoxicity. J. Control. Release.

[5] Ninomiya K., Kawabata S., Tashita H., Shimizu N. (2014). Ultrasound-mediated drug delivery using liposomes modified with a thermosensitive polymer. Ultrason. Sonochem..

[6] Ding J.X., Chen J.J., Li D., Xiao C.S., Zhang J.C., He C.L., Zhuang X.L., Chen X.S. (2013). Biocompatible reduction-responsive polypeptide micelles as nanocarriers for enhanced chemotherapy efficacy* in vitro*. J. Mater. Chem. B.

[7] Liang J., Wu W.L., Xu X.D., Zhuo R.X., Zhang X.Z. (2014). pH responsive micelle self-assembled from a new amphiphilic peptide as anti-tumor drug carrier. Colloid. Surf. B.

[8] Ding J.X., Shi F.H., Li D., Chen L., Zhuang X.L., Chen X.S. (2013). Enhanced endocytosis of acid-sensitive doxorubicin derivatives with intelligent nanogel for improved security and efficacy. Biomater. Sci..

[9] Nukolova N.V., Oberoi H.S., Cohen S.M., Kabanov A.V., Bronich T.K. (2011). Folate-decorated nanogels for targeted therapy of ovarian cancer. Biomaterials.

[10] Kobayashi H., Watanabe R., Choyke P.L. (2014). Improving conventional enhanced permeability and retention (EPR) effects; What is the appropriate target?. Theranostics.

[11] Gou P.F., Liu W.W., Mao W.W., Tang J.B., Shen Y.Q., Sui M.H. (2013). Self-assembling doxorubicin prodrug forming nanoparticles for cancer chemotherapy: Synthesis and anticancer study* in vitro* and* in vivo*. J. Mater. Chem. B.

[12] Kamimura M., Furukawa T., Akiyama S.-i., Nagasaki Y. (2013). Enhanced intracellular drug delivery of pH-sensitive doxorubicin/poly(ethylene glycol)-*block*-poly(4-vinylbenzylphosphonate) nanoparticles in multi-drug resistant human epidermoid KB carcinoma cells. Biomater. Sci. UK.

[13] Tang Z.H., Chen X.S., Yang Y.K., Pang X., Sun J.R., Zhang X.F., Jing X.B. (2004). Stereoselective polymerization of *rac*-lactide with a bulky aluminum/Schiff base complex. J. Polym. Sci. Pol. Chem..

[14] Zhao Z.W., Yao X.M., Zhang Z., Chen L., He C.L., Chen X.S. (2014). Boronic acid shell-crosslinked dextran-*b*-PLA micelles for acid-responsive drug delivery. Macromol. Biosci..

[15] Li Y., Lin J.Y., Wu H.J., Jia M.M., Yuan C.H., Chang Y., Hou Z.Q., Dai L.Z. (2014). Novel methotrexate prodrug-targeted drug delivery system based on PEG-lipid-PLA hybrid nanoparticles for enhanced anticancer efficacy and reduced toxicity of mitomycin C. J. Mater. Chem. B.

[16] Serra T., Planell J.A., Navarro M. (2013). High-resolution PLA-based composite scaffolds *via* 3-D printing technology. Acta. Biomater..

[17] Lu Y.M., Cheng L.M., Pei G.X., Cai Z., Pan L., Su J., Zhang K.H., Guo L.L., Yu Q.S., Guo Y.R. (2013). Experimental study of repairing femoral bone defects with nHA/RHLC/PLA scaffold composite with endothelial cells and osteoblasts in canines. Chin. Med. J..

[18] Kulkarni R.K., Pani K.C., Neuman C., Leonard F. (1966). Polylactic acid for surgical implants. Arch. Surg..

[19] Bradley A.J., Murad K.L., Regan K.L., Scott M.D. (2002). Biophysical consequences of linker chemistry and polymer size on stealth erythrocytes: Size does matter. BBA Biomembr..

[20] Kim K., Yu M., Zong X.H., Chiu J., Fang D.F., Seo Y.S., Hsiao B.S., Chu B., Hadjiargyrou M. (2003). Control of degradation rate and hydrophilicity in electrospun non-woven poly(D,L-lactide) nanofiber scaffolds for biomedical applications. Biomaterials.

[21] Gref R., Luck M., Quellec P., Marchand M., Dellacherie E., Harnisch S., Blunk T., Muller R.H. (2000). ‘Stealth’ corona-core nanoparticles surface modified by polyethylene glycol (PEG): Influences of the corona (PEG chain length and surface density) and of the core composition on phagocytic uptake and plasma protein adsorption. Colloid. Surface B.

[22] Klibanov A.L., Maruyama K., Torchilin V.P., Huang L. (1990). Amphipathic polyethyleneglycols effectively prolong the circulation time of liposomes. FEBS Lett..

[23] Riley T., Stolnik S., Heald C.R., Xiong C.D., Garnett M.C., Illum L., Davis S.S., Purkiss S.C., Barlow R.J., Gellert P.R. (2001). Physicochemical evaluation of nanoparticles assembled from poly(lactic acid)-poly(ethylene glycol) (PLA-PEG) block copolymers as drug delivery vehicles. Langmuir.

[24] Heald C.R., Stolnik S., De Matteis C., Garnett M.C., Illum L., Davis S.S., Leermakers F.A.M. (2003). Characterisation of poly(lactic acid): Poly(ethyleneoxide) (PLA:PEG) nanoparticles using the self-consistent theory modelling approach. Colloid. Surf. A.

[25] Fu C.H., Sun X.L., Liu D.H., Chen Z.J., Lu Z.J., Zhang N. (2011). Biodegradable tri-block copolymer poly(lactic acid)-poly(ethylene glycol)-poly(L-lysine)(PLA-PEG-PLL) as a non-viral vector to enhance gene transfection. Int. J. Mol. Sci..

[26] Ocal H., Arica-Yegin B., Vural I., Goracinova K., Calis S. (2014). 5-fluorouracil-loaded PLA/PLGA PEG-PPG-PEG polymeric nanoparticles: Formulation,* in vitro* characterization and cell culture studies. Drug Dev. Ind. Pharm..

[27] Mukose T., Fujiwara T., Nakano J., Taniguchi I., Miyamoto M., Kimura Y., Teraoka I., Lee C.W. (2004). Hydrogel formation between enantiomeric B-A-B-type block copolymers of polylactides (PLLA or PDLA: A) and polyoxyethylene (PEG: B); PEG-PLLA-PEG and PEG-PDLA-PEG. Macromol. Biosci..

[28] Hiemstra C., Zhong Z., Li L., Dijkstra P.J., Feijen J. (2006). *In-situ* formation of biodegradable hydrogels by stereocomplexation of PEG-(PLLA) 8 and PEG-(PDLA) 8 star block copolymers. Biomacromolecules.

[29] Fujiwara T., Mukose T., Yamaoka T., Yamane H., Sakurai S., Kimura Y. (2001). Novel thermo-responsive formation of a hydrogel by stereo-complexation between PLLA-PEG-PLLA and PDLA-PEG-PDLA block copolymers. Macromol. Biosci..

[30] Ouahab A., Cheraga N., Onoja V., Shen Y., Tu J.S. (2014). Novel pH-sensitive charge-reversal cell penetrating peptide conjugated PEG-PLA micelles for docetaxel delivery: *In vitro* study. Int. J. Pharm..

[31] Chen L., Xie Z.G., Hu J.L., Chen X.S., Jing X.B. (2007). Enantiomeric PLA–PEG block copolymers and their stereocomplex micelles used as rifampin delivery. J. Nanopart. Res..

[32] Liu D.H., Ding J.X., Xu W.G., Song X.F., Zhuang X.L., Chen X.S. (2014). Stereocomplex micelles based on 4-armed poly(ethylene glycol )-polylactide enantiomeric copolymers for drug delivery. Acta Polym. Sin..

[33] Lee J.L., Ahn J.H., Park S.H., Lim H.Y., Kwon J.H., Ahn S., Song C., Hong J.H., Kim C.S., Ahn H. (2012). Phase II study of a cremophor-free, polymeric micelle formulation of paclitaxel for patients with advanced urothelial cancer previously treated with gemcitabine and platinum. Invest. New Drug..

[34] Ahn H.K., Jung M., Sym S.J., Shin D.B., Kang S.M., Kyung S.Y., Park J.W., Jeong S.H., Cho E.K. (2014). A phase II trial of cremorphor EL-free paclitaxel (Genexol-PM) and gemcitabine in patients with advanced non-small cell lung cancer. Cancer Chemoth. Pharm..

[35] Simons K., Ikonen E. (2000). Cell biology—How cells handle cholesterol. Science.

[36] Lee A.L.Z., Venkataraman S., Sirat S.B.M., Gao S.J., Hedrick J.L., Yang Y.Y. (2012). The use of cholesterol-containing biodegradable block copolymers to exploit hydrophobic interactions for the delivery of anticancer drugs. Biomaterials.

[37] Pucadyil T.J., Chattopadhyay A. (2006). Role of cholesterol in the function and organization of G-protein coupled receptors. Prog. Lipid Res..

[38] Laskar P., Samanta S., Ghosh S.K., Dey J. (2014). *In vitro* evaluation of pH-sensitive cholesterol-containing stable polymeric micelles for delivery of camptothecin. J. Colloid Interface Sci..

[39] Yang B., Lv Y., Zhu J.Y., Han Y.T., Jia H.Z., Chen W.H., Feng J., Zhang X.Z., Zhuo R.X. (2014). A pH-responsive drug nanovehicle constructed by reversible attachment of cholesterol to PEGylated poly(L-lysine) via catechol-boronic acid ester formation. Acta Biomater..

[40] Cameron D.J.A., Shaver M.P. (2011). Aliphatic polyester polymer stars: Synthesis, properties and applications in biomedicine and nanotechnology. Chem. Soc. Rev..

[41] Ding J.X., Xu W.G., Zhang Y., Sun D.K., Xiao C.S., Liu D.H., Zhu X.J., Chen X.S. (2013). Self-reinforced endocytoses of smart polypeptide nanogels for “on-demand” drug delivery. J. Control. Release.

[42] Liu L., Li C.X., Li X.C., Yuan Z., An Y.L., He B.L. (2001). Biodegradable polylactide/poly(ethylene glycol)/polylactide triblock copolymer micelles as anticancer drug carriers. J. Appl. Polym. Sci..

[43] Kang N., Perron M.E., Prud’homme R.E., Zhang Y.B., Gaucher G., Leroux J.C. (2005). Stereocomplex block copolymer micelles: Core-shell nanostructures with enhanced stability. Nano Lett..

[44] Li M.Q., Tang Z.H., Lv S.X., Song W.T., Hong H., Jing X.B., Zhang Y.Y., Chen X.S. (2014). Cisplatin crosslinked pH-sensitive nanoparticles for efficient delivery of doxorubicin. Biomaterials.

[45] Lee M., Rentz J., Han S.O., Bull D.A., Kim S.W. (2003). Water-soluble lipopolymer as an efficient carrier for gene delivery to myocardium. Gene Ther..

[46] Maxfield F.R., Meer G.V. (2010). Cholesterol, the central lipid of mammalian cells. Curr. Opin. Cell Biol..

[47] Dai J., Lin S.D., Cheng D., Zou S.Y., Shuai X.T. (2011). Interlayer-crosslinked micelle with partially hydrated core showing reduction and pH dual sensitivity for pinpointed intracellular drug release. Angew. Chem. Int. Edit..

[48] Sun D.K., Ding J.X., Xiao C.S., Chen J.J., Zhuang X.L., Chen X.S. (2014). Preclinical evaluation of antitumor activity of acid-sensitive PEGylated doxorubicin. ACS Appl. Mater. Inter..

[49] Ding J.X., Zhao L., Li D., Xiao C.S., Zhuang X.L., Chen X.S. (2013). Thermo-responsive “hairy-rod” polypeptides for smart antitumor drug delivery. Polym. Chem..

[50] Wang Y., Wang H.B., Liu G.Y., Liu X.S., Jin Q., Ji J. (2013). Self-assembly of near-monodisperse redox-sensitive micelles from cholesterol-conjugated biomimetic copolymers. Macromol. Biosci..

[51] Benival D.M., Devarajan P.V. (2012). Lipomer of doxorubicin hydrochloride for enhanced oral bioavailability. Int. J. Pharm..

